# Dancing together in virtual space: experiential and agentive dimensions of perceptual crossing support microactivities and dynamic embodiment in social cognition

**DOI:** 10.1007/s10339-025-01306-4

**Published:** 2025-11-22

**Authors:** Johannes Wagemann, Leonardo Zapata-Fonseca, Stephen Estelle, Tom Froese

**Affiliations:** 1 Institute for Waldorf Education, Inclusion and Interculturalism, Alanus University, Campus Mannheim, Am Exerzierplatz 21, D–68167 Mannheim, Germany; 2 Embodied Cognitive Science Unit, Okinawa Institute of Science and Technology Graduate University (OIST), Okinawa, Japan

**Keywords:** Social cognition, Social interaction, Embodiment, First-person methodology, Action phases, Microactivities

## Abstract

The Perceptual Crossing Paradigm (PCP) is one of the most radical settings in social cognition research, as it confines dyadic encounter to one-dimensional movements in a virtual space and haptic feedback via human-computer interfaces. While the PCP has already led to insightful results in different settings and populations, the first-person perspective of participants on their experience and agency has not yet been systematically investigated. However, to understand the precise mechanisms by which test partners interact and identify each other, and the role that embodiment plays in this, behavioral data must be complemented by qualitative first-person data. Therefore, our PCP study (*N*=54) included open-ended self-reports collected over six trials which were analyzed with a mixed-methods approach using qualitative coding and different forms of quantification and statistical tests. Qualitatively, the 62 codes organized in hierarchical levels provide a fine-grained picture of individual experience (e.g., fourteen emotions) and interindividual aspects of agency (action phases, proactive and receptive microactivities originating from both partners). Unexpectedly, in quantitative regard, code frequencies of all four microactivities were significantly higher in this minimalistic setting compared to other social cognition studies with more natural settings. And their distribution across the action phases (intention, action execution, evaluation) yielded a significant pattern that can be explained by differentiating physical and mental actions as stronger and weaker forms of embodiment, supporting a dynamically embodied concept of social interaction.

## Introduction

Since its invention, the perceptual crossing paradigm (PCP) provides one of the most radical empirical settings for exploring the fundamental conditions of social cognition (Auvray et al. 2009; Lenay et al. 2006). Two individuals move and interact via human-computer interfaces in a one-dimensional virtual space only by haptic feedback without other (e.g., visual, auditory) forms of sensory or informational contact. In addition, the subjects must distinguish their test partners from other static and moving (the so-called shadow) objects in the virtual environment to be able to recognize them safely. As the PCP was developed within the enactive approach to cognitive science to extend the “classical” but one-sided, computational, representational, and neurocentric view (Newen et al. 2018; Pylyshyn 1984), it should clarify the roles of interaction and embodiment for social cognition. While interaction shifts the focus from mechanisms within individuals (e.g., neural activity) to processes unfolding between them, embodiment highlights sensory-motor coupling in different modalities and resonance between the whole, enacted bodies involved in dyadic settings. Therefore, the PCP experimentally simplifies the natural conditions of social cognition through restricting the options of sensory perception and bodily behavior to a limited set of controllable conditions.

As a first result, several PCP studies demonstrated that people are well able to reliably identify their test partner under the outlined constraints (Auvray et al. 2009; Auvray and Rohde 2012), even if they only have one minute for this task (Froese et al. 2014a). Another crucial finding was that successful identification of the partner was correlated with interactively coordinated turntaking behavior in which participants complementarily changed between actively stimulating the other and passively waiting for stimulation by the other (Kojima et al., 2017). Other studies explored the PCP for different populations, including adolescents and a clinical sample (Hermans et al., 2020; Zapata-Fonseca et al. 2018), and there are also studies that have developed alternative versions by involving artificial agents based on different behavioral models (Rohde and Di Paolo 2008), or by extending the behavioral variables through physiological measurement (e.g., electroencephalogram, electrocardiogram, electrodermal activity) and psychometric data, i.e., personality traits (Lerique et al., 2024). However, what has not yet been systematically and comprehensively investigated in the PCP is participants’ first-person perspective on their lived experience and agency, as well as the relationship between such qualitative data and externally measurable variables. In the context of enactivism, the inclusion of qualitative first-person data is a desideratum, since both interaction and embodiment do not possess only externally measurable aspects but also internal ones, which are only accessible through introspection. First, as interaction includes action which, in a psychological context, refers to what motivated, conscious agents intend, execute, and evaluate, it cannot be reduced to externally measured behavior including neural activity (Pacherie 2008). In cognitive science, subjective or phenomenal agency, which can directly only be studied via qualitative first-person data, must also be distinguished from objective agency, as the latter is about the functional architecture of agency, i.e., what a system does and how it appears to act in an organized, autonomous way, regardless of whether it feels like anything from the inside (Gallagher 2000; Synofzik et al. 2008). To analyze the phenomena of subjective agency, we refer to the proven Rubicon model of action phases (Heckhausen and Gollwitzer 1987) and its extension to the mindset theory of action phases (Gollwitzer 1990, 2012) as a blueprint for category formation in qualitative analysis. Second, beyond the obvious fact that human agents have bodies and, in this sense, perform embodied (inter)actions, there is a difference between their experience of having a body and being a body, for example (Plessner 2019), pointing to experientially distinct, more or less distanced modes of embodiment. Since the inception of enactivism and its extension to 4E cognition, phenomenology and experience have always been a reference point, which is why they should also be included now.

Having said this, there are some PCP studies that collected and analyzed qualitative self-reports to a limited extent. To begin with, Froese and colleagues (2014a) found that joint success trials are generally associated with reports of clearer social awareness than individual successes or wrong responses. While this key finding was subsequently replicated by two follow-up studies (Froese et al. 2020; Lerique et al., 2024), an exploratory diachronic analysis of subjective reports was also conducted for the aforementioned study (Froese et al. 2014b). Finally, in Lenay’s (2010) philosophical analysis of the “feeling” of perceptual crossing, which draws on the study by Auvray and colleagues (2009), notable metaphors of kinesthetic interaction such as shaking hands, dancing, and cuddling were reported. While these are interesting initial insights into the lived experience and agency of participants, they do not provide a comprehensive account of these dimensions and leave many questions open, particularly in relation to interactivity and embodiment.

Although not in a PCP framework, studies on social cognition have already been conducted in more natural or direct settings with qualitative first-person data, which also address interactivity and embodiment. Using tasks with changing (Wagemann & Weger, 2021) or stable dyads (Wagemann et al., 2022), written self-reports, qualitative content analysis, intercoder reliability tests, quantification of code frequencies and statistical tests, the latter study was able to explain interactivity as an oscillation between interpersonal and personal, individualistic modes of experience. Both studies revealed that interaction can be traced back to a dynamic pattern of microactivities, congruent to the above-mentioned turntaking behavior, but additionally with a fine-grained differentiation of proactive-focusing and receptive-opening gestures emerging from both the person reporting and the person interacting with them. The indicated qualitative, multimethod approach was also applied to the action phases within the Rubicon model in one of our arts-based research studies (Wagemann & Starosky, 2024). Hence, while the Rubicon model provides a scaffold of temporal and functional phases, microactivities in social cognition represent a partial but significant content of them. Regarding embodiment in social cognition, Wagemann & Weger (2021) found a few statements about transparency of the physical appearance of the dyadic partner that was stronger for the experimental condition in which subjects were forbidden to touch each other physically, although the difference to the other condition with allowed physical contact was not statistically significant. With a refined methodology, our 2022 study could show that body-related experience is significantly stronger associated with proactive-focusing activity, whereas the contrary is true for receptive-opening activity.

Here, these two lines of research are brought together by collecting not only behavioral and psychometric data but also comprehensive qualitative self-reports in a PCP setting and examining these by the mixed-methods analyses indicated. As there is a natural division between the different data types and suitable forms of analysis, in this article we only present the results and conclusions based on the qualitative data. The other data will be released alongside this article and could be the subject of future analysis. The following samples from three data sets suggest that it is indeed worth devoting an entire article exclusively to the qualitative data: “At the beginning, I felt an insecurity inside me that I had to actively push back. I asked myself whether it was even possible to find the other person’s avatar. During the experiment, I experienced moments in which I had the impression that I was moving in unison with my experiment partner. These moments felt warm and safe.”, “I tried to “catch” the person like in a game and then run away again myself. I waited a bit for myself to be caught, but I was the catcher in this round”, “Very intensive contact. We moved together for a while, like leading and being led at the same time. I thought it felt a bit like a ‘blind dance’”.

## Method

### Participants and materials

The study was conducted in the first half of March 2024 at Alanus University, Campus Mannheim. Participants were recruited at the university through information posters and emails to all Alanus students and inhabitants of the student residence. In addition, participation was advertised in some courses with short presentations and flyers. In sum, fifty-four persons (42 females, 12 males) between 18 and 53 years (*M* = 26.1) participated in the study and received 26 euros as an expense allowance for the maximum duration of a trial of 1.5 h. Participants could register individually or in pairs; in the former case, they were divided into dyads at random or according to the scheduling options they indicated. A few days before the trial, participants were sent the instructions by email and asked to read them in advance. This was combined with written informed consent and an initial screening asking participants to disclose any psychiatric or neurological conditions and use of psychoactive medication (e.g., antidepressants, antiepileptics, antipsychotics), as this could potentially affect their experience and performance during the study. One participant reported taking Quetiapin, Sertraline, and Aripiprazol (AK05), another one Citalopram (AD02); but in lack of specific hypotheses, we included both of them and reserve the right to examine this aspect in further evaluation steps. At the beginning of each trial, participants were welcomed pairwise and asked to read through the instructions again carefully and ask any questions they had which were answered by one of the experimenters. However, neither before nor during data collection participants were informed of possible strategies to be deployed or phenomena that might occur during the trial and potential hypothetical explanations. Along with the demographic data (age, gender and nationality) collected via the software system, participants were asked about the relationship status with their team partner, choosing between the following options: (1) I don’t know the person or only know them by sight, (2) I know the person from joint courses, (3) I am friends with the person, (4) For me, this person is like family. Then, before the start of the perceptual crossing experiment, participants completed the Big Five personality questionnaire with 60 items (German version of BFI-2, Soto and John 2017). During the experimental procedure after each trial, they responded to three four-point Likert-scale items, the last one of which included the adapted Perceptual Awareness Scale (PAS) as described by Froese and colleagues (2014a): (a) “How often did you experience the presence of your partner during the entire trial?” (b) “How strong was the feeling that you did something together with your partner” (c) “How clearly did you experience the presence of your partner immediately before pressing the button?” As mentioned above, all these quantitative data were not included in the analyses presented in this article but will be considered in its second part.

### Apparatus

**Fig. 1:**
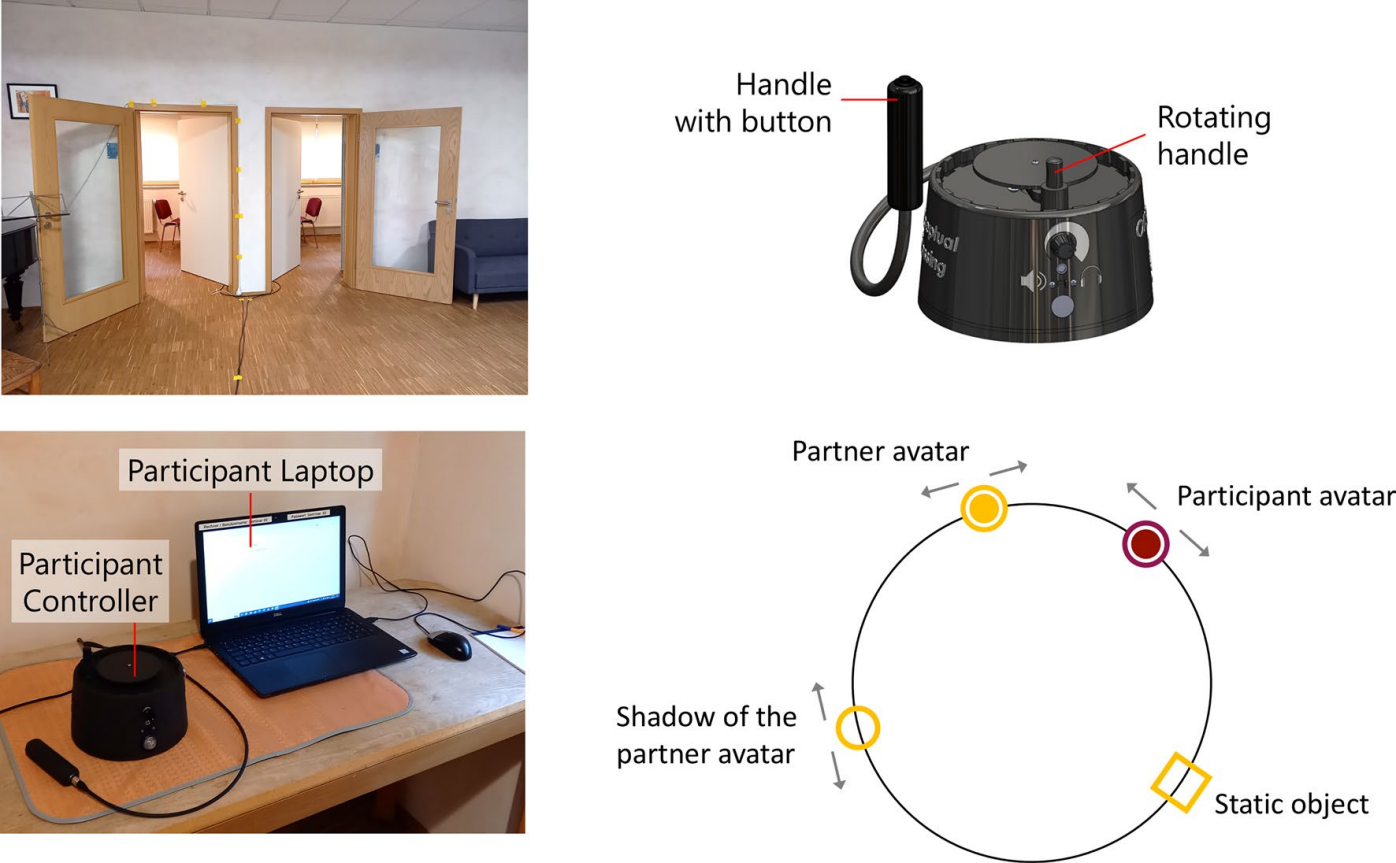
Rooms, setting, participants controller and virtual space.

Participants were seated in separate, adjacent rooms with closed doors (Fig. 1). The apparatus for each participant comprises two components: participant controller and participant laptop. The controllers enable interaction within a virtual space, delivering both haptic feedback and audio cues to users for orientation during the test sequence, they are connected to a central console managing data processing, transmission, and synchronization between the controllers and participant laptops. Each controller includes a rotating handle, capable of continuous rotation on the top surface, which facilitates interaction between participants. Additionally, a handle with an integrated button allows participants to signal when they think they have found their partner avatar. Inside both the rotating handle and the button handle, motors are embedded to provide haptic feedback via vibration. Through laptops, participants are guided through the test procedure and enter their quantitative and qualitative responses. More in-depth information on the technical system can be found in Estelle and colleagues (2024).

As in other PCP studies (e.g., Froese et al., 2014), the virtual space consists in a closed circle and is populated by different, movable and static objects (Fig. 1). From the participants’ perspective, their avatar can interact with a static object, the partner avatar, and its shadow. The shadow of the partner avatar always moves synchronously with it at a constant distance of 90°. At the beginning of each trial, participants’ avatars are placed at 180° from each other; the static object is placed randomly, but always at 180° from the partner’s static object to avoid situations that are too ambiguous. Importantly, overlaps of the participant avatar with all three other objects leads to the same kind of vibration feedback. This information is part of the instructions which will be explained in the next section.

### Procedure

After collecting the demographic and personality data, participants underwent a training phase consisting of two one-minute trials. The first trial was conducted with the screen visible, allowing participants to familiarize themselves with the virtual environment, objects, and their movements. The second trial was performed with a blackened screen to simulate the conditions of the actual experimental trials. For both the training and the regular trials, participants were instructed (a) to find their partner’s avatar, (b) to help them to find their own avatar and (c) to press the button when they think they have found the partner avatar. Participants were permitted to keep their eyes open or to close them, as they preferred. Following the training phase, participants completed a first block of eight one-minute trials. After each trial within this block, they responded to the Likert-scale items, as described above. After the initial eight trials, participants proceeded to a second block consisting of six one-minute trials, in which they completed the same Likert-scale items and also responded to open-ended questions regarding the strategies and experiences they employed during each trial (see next section). After these two blocks comprising 14 trials in total with their respective questionnaires, participants answered a final open-ended question about their overall strategy, bringing the experiment to an end. The restriction of qualitative data collection to the last six trials can be justified by reasons of participants’ training and familiarization with the task, their limited concentration during the more than one hour procedure, and the reduction in redundancy in the qualitative data.

### Qualitative instrument: open-ended questionnaire

For collecting the qualitative data, open-ended questionnaires were to be answered after each of the last six trials. This choice can be seen as a well-balanced compromise between the qualitative granularity necessary for an in-depth analysis of first-person experience, on the one hand, and a pragmatic collection of data for each individual trial, on the other hand. In addition, this method has been proven in other contexts (e.g., Wagemann & Walter, 2024) and avoids the risk of different biases such as social interaction and experimenter expectation (Adams-Quackenbusch et al. 2019; Salazar 1990). To suggest as little content as possible and to capture as wide a range of experiences as possible, the following two questions were asked: (1) “What did you experience during this trial and what did you try to do to solve the task?” (2) “When you pressed the button: What prompted you to press the button immediately before you pressed it? If you did not press the button: Why did you not press it?” While the first question addresses experience and agency in a quite general way, the second question focuses these aspects on the moment of the button press, if applicable. Both questions are intended to deliver fine-grained first-person data but also to be related in detail to the different aspects of the behavioral data.

### Observational and experimental design

As this is the first PCP study to comprehensively examine qualitative aspects of experience and agency, we focus on its exploratory nature before proposing hypotheses that would need to be tested in an experimental design or by including the quantitative data collected. Nevertheless, different forms of quantification of the qualitative data allow them to be treated at least as quasi-independent (or predictor) and dependent (or outcome) variables in the context of our observational (or non-experimental) design. One option is to code the same qualitative data according to different theoretical models. While, in this case, the code frequencies of the more established theory (e.g., mindset theory of action phases) serve as quasi-independent variables, those of the more hypothetical theory (e.g., microactivities) take the role of dependent variables, as will be explained in more detail below. Apart from this, this study even offers experimental dimensions, as the code frequencies of cross-study qualitative categories (e.g., microactivities) can be compared with those of our previous studies on social cognition. Here, the different empirical settings establish the independent variable. Another experimental dimension will be pursued regarding code frequencies pertaining to qualitative variables (e.g., agency with accumulated action phases) analyzed for the three different objects (static object, shadow, partner avatar) the latter of which represent independent variables, as they are incorporated in the task setting in the same way for all trials and participants.

### Data analysis

**Fig. 2:**
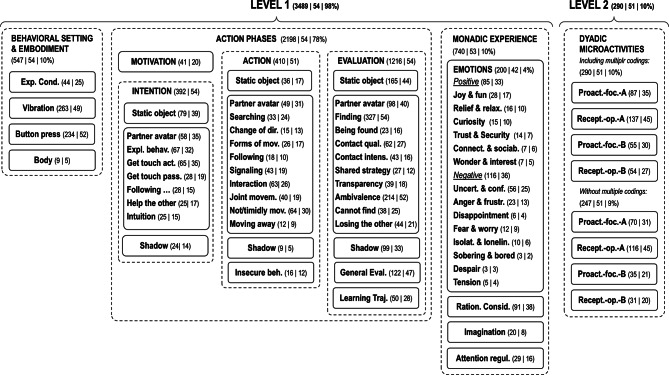
Structured category system and coding levels (*N* = 54). Numbers indicate how many segments were coded in how many data sets (participants) according to a certain code. Percentage show the data coverage by codes on text character

For data analysis, we used a multimethod approach with various qualitative and quantitative aspects, as developed by the task-based introspective inquiry (Wagemann & Starosky, 2024; Wagemann & Walter, 2024). Qualitative data is first analyzed in terms of a qualitative content analysis, with whole sentences or partial sentences down to single words serving as coding units. In most cases, the categories are determined in such a way that there is no overlap between the codes of a level. Where relevant overlaps occur, this is pointed out separately, as in the case of microactivities, for example, where two code sets are distinguished: one with multiple coding and one without (Fig. 2). Second, the results of the qualitative analysis are quantified in different ways; and, in addition, quantitative measures are derived from the qualitative data independently of the qualitative analysis. This spectrum of “early” and “late” forms of quantification can be seen as the major methodological innovation of this PCP study providing access to lived experience and agency without neglecting alignment with proven quality criteria of quantitative cognitive and behavioral research. Essentially, this approach aims to overcome incommensurability problems that can occur in traditional mixed methods studies, where different types of data (qualitative and quantitative) are collected and analyzed separately before the results are integrated (Creswell 2009; Small 2011). With early and late forms of quantification of qualitative data or results, bridges can be built that also promise better integration with the purely quantitative data collected in this study.

First, in terms of early quantification, some quantitative aspects of the qualitative text data were analyzed independently of the qualitative analysis such as the protocol length in words and the proportion of first-person pronouns (singular, plural), since this allows for assessing the methodological validity of the data and can be also used as dependent variables. Next, the qualitative content analysis of the text data was conducted with an integrative bottom-up/top-down approach (Hsieh and Shannon 2005; Mayring 2000) leading to a fine-grained hierarchical category system covering all aspects of experience and agency reported in the protocols. Concretely, the category system (Fig. 2) and its codebook (Table 1) emerged by reading through the data, defining categories with which text segments could be coded, and refining and structuring the categories in iterative steps. As text segments to be coded whole or partial sentences were used in most cases, whereas for some categories even single words were coded (e.g., for “vibration” or “knob pressing”; Miles et al. 2020). As indicated in terms of bottom-up/top-down analysis, this procedure dynamically integrates data-driven or inductive and theory-driven or deductive generation of codes/categories. It should be noted that the bottom-up analysis does not preclude the use of appropriate categories from our previous studies in cognitive science contexts, adapted as necessary to the current task and data. In contrast, top-down analysis introduces codes that are derived from coherent and empirically supported theories, whereby, of course, this does not rule out the need to adapt the definitions to the current context. Similarly, as in previous studies conducted with the same methodology, we structure the category system in two hierarchical levels serving different purposes in view of the research questions. Level 1 is intended to demonstrate that the data can be completely and consistently coded with both bottom-up and top-down aspects to prevent the suspicion of cherry picking. Beyond this, Level 1 comprises important facets and structures of first-person experience and agency complementing the purely behavioral dimensions of perceptual crossing. Level 2 focuses in a top-down manner on microactivities as a more specific and subtle aspect of social cognition investigated in two previous studies (Wagemann & Weger, 2021; Wagemann et al. 2022), which, as expected, cover a smaller part of the data and would not be apparent without a targeted search.

**Table 1:** **First and second coding level**. Categories with subcategories, short descriptions, and exemplary excerpts from the data

Level 1	Description	Examples
BEHAVIOR, ACTION, AND EXPERIENCE	*Level 1 codes were created in the first step of the analysis through an integrative approach that alternated between open bottom-up coding and top-down application of established concepts or theories*	
1. BEHAVIORAL SETTING AND BODY	*These codes comprise experiences referring to the external test setting, stimulus and response mediated by the PC device, and participants’ own body*	
1.1 Test Setting	Test situation, time experience/management, sequence of test phases	"… but then the time was up." “I would have needed a few more seconds …” "Writing brought me out a bit, I started to think more and my feelings were no longer so free."
1.2. Vibration	The fact and duration or experienced quality of the vibration feedback is described with “vibration” or corresponding words, often only a single word is coded	"…when a vibration passes me by." "…and a moment of permanent vibration occurred." "… the whirring …"
1.3 Button Press	The fact and temporal or psychological context of pressing the button (or not) is described	“Just as I was about to decide to press the button …” “This time, I pressed too hastily.” “I did not press, because …”
1.4 Body	It is described how certain parts of the body are used (hand or finger to use the wheel, eyes closed or opened). Bodily sensations of any kind were not mentioned	“Moving the wheel by fingertip only.” “I had my eyes closed at first and then opened them during the process.”
2. ACTION PHASES	*These codes are structured according to the Rubicon model and mindset theory of action phases*	
2.1 Motivation	Motivations initiate, sustain, and direct intentional behavior through the reasons or desires that drive participants to pursue their goals. Intrinsic and extrinsic motivations influence decision-making during the predecisional phase and therefore might be less explicit during the action and evaluation phases. For example, they can be expressed as higher or lower levels of motivation, specific desires, eagerness, ambition, drive, spur, (im)patience, questions about purpose, challenge	“I wanted to turn it into a game and not just wait until we had found each other.” “A strong desire for exchange …” “I also have more of an ambition to do it quickly.”
2.2 Intention	*Intentions are commitments to achieving a particular (sub)goal by representing action plans, strategies or strategy changes. Participants mention which (sub)goals they try to achieve by which means. (Sub)goals and means or strategies include external routines or movements or internal attitudes (e.g., exploration, systematic approach, openness, intuition). Intentions can refer to individual trials or the overall test and can be distinguished in their relation to the static object, the partner avatar and its shadow*	
2.2.1 Static Object	The subgoal of identifying and locating the static object is mentioned and sometimes also justified	“I tried to find out where the static element is located.” “I also looked for my fixed station here first to rule it out.”
2.2.2 Partner Avatar	One or different strategies, systematic approaches, or attitudes – or lack thereof – are mentioned in the context of the main goal of finding the partner avatar, which is mostly implicit with this code	“… and had no idea how to find her, let alone how to make myself noticeable to her.” “I had resolved to move my avatar more slowly.” “I followed through with my previous strategy.”
Explore behavior / space	Participants attempt to explore the behavior or reactions of occurring vibrations or to obtain general orientation in the virtual space	“…to see if the vibration stops…” “I tried to get my bearings …”
Get in touch (active)	The goal or attempt to find the partner avatar and get in touch is explicitly mentioned and can be complemented by an active approach or attitude	“… to find my counterpart.” “ But I have often actively tried to find the person.”
Get in touch (passive)	Participants express a more passive, wait-and-see attitude and the intention to be found and identified by their partner	“… so that the other person can find me and distinguish me from the static object.” “… to see if she stops or comes back to me …”
Keep in touch / follow	Participants try to keep contact with their partner or follow them	“… I tried to keep in touch for as long as possible…” “I always tried to follow my partner and stay close to her.”
Help the other	Participants intend to help their partner to find them by keeping contact or through specific attitudes/approaches	“… and then to help my partner find me…” “When I found the other person, I tried to help her by moving slowly around the circle.”
Intuition	Participants mention a general attitude or change in strategy to rely on their gut feeling or intuition	“Overall, I just relied on my intuition.” “…to approach things less cerebrally and more with a gut feeling.”
2.2.3 Shadow	Participants explicitly mention their intention to identify the shadow, distinguish it from the partner avatar, or implicitly include the shadow by referring to moving objects	“…to make sure it wasn't her shadow I was chasing or had found.” “… and tried to find out if she also communicates with me, and that it’s not the shadow.”
2.3 Action	*Actions are concrete operations or routines performed by the participants to achieve the goals inherent in their intentions. They are described in terms of movement patterns or the execution of specific steps of routines. Unsecure behavior is also included. Actions can be distinguished in their relation to the static object, the partner avatar and its shadow*	
2.3.1 Static Object	The static object is explicitly mentioned as the goal or object of action	“I checked several times where the object was …” “Then I set off in search of the object.”
2.3.2 Partner Avatar	All actions or explorations are coded that either explicitly refer to the partner avatar, or interactions, or are rather unspecific but performed in the context of the task	“It took about three quarters of a lap on the circular track …” “I tested whether the location and duration of the vibration changed.”
2.3.2.1 Searching	Participants search for the other person/avatar or scan the virtual space	“…I set out in search of the partner.” “… then searched for the moving elements”
2.3.2.2 Change of direction	Participants perform a change of direction (back and forth, up and down) or move on the spot	“So, I went through the circle again and again, also in both directions …” “… I moved slightly up and down with each additional vibration …”
2.3.2.3 Forms of movement	Forms of movement: speed/tempo (fast, slow, slight, constant, reduced, increased), uniform, or smooth movement)	“… by moving slowly across the circle.” “… I tried to be faster than her by moving at a faster pace”
2.3.2.4 Following and returning	The partner seemed to have been found once and is subsequently, followed, pursued, traced, tried to be caught. Returning to promising positions is also part of this code	“I 'stuck to her heels', so to speak.” “I then moved over and over again on the affected areas.”
2.3.2.5 Signaling	Decidedly signaling character, attracting the attention of the other: nudging, docking, walking/running against the other, give a signal, let them know, even if it comes from the other person	“We then always stopped and signaled to the other person that it was us by walking back and forth.” “We gave each other the wiggle signal.”
2.3.2.6 Interaction	All kinds of interaction or communication, possibly without further specification	“…and finding our own communication.” “We felt our way towards each other and touched each other again and again …”
2.3.2.7 Joint Movement	All expressions that indicate a joint movement with the other avatar	“But if he stayed longer or moved then with me …” “It is most awesome when we walk in the same direction together.”
2.3.2.8 Not / Timidly Moving	Slowed or delayed movement or even standstill	“… and waited longer in one place …” “I tried to move very little or not at all during this test run.”
2.3.2.9 Moving Away	Leaving the partner avatar or the static object to find the partner avatar	“Once, I walked a bit away from our contact point …” “ … or I have “fled”.”
2.3.3 Shadow	The shadow is explicitly mentioned as the goal or object of action	„…Intensified search for the shadow, or its reaction.” “I made several more trips towards the end [to feel her shadow …]”
2.3.4 Insecure Behavior	Unplanned, hectic, and aimless action without a clear, systematic approach	“Seemingly without a strategy, just try it out and see.” “I would describe my approach as improvised and indecisive.”
2.4 Evaluation	*Evaluations refer to the outcomes of performed actions and deployed strategies and reflect them in terms of success and failure including experiential qualities and self-assessment of participants’s own performance and effort. Evaluations can refer to individual trials or the overall requirements and execution of the test and can also reflect on learning effects. They can be distinguished in their relation to the static object, the partner avatar and its shadow*	
2.4.1 Static Object	The static object is explicitly or indirectly (but clearly) mentioned as the reference point of evaluation, including qualitative experiences	”… weil ich mir ziemlich sicher bin, dass es das Objekt war, bei welchem ich den Knopf gedrückt habe.” “… weil ich kein klares statisches Element lokalisieren konnte.“
2.4.2 Partner Avatar	The way and intention participants evaluate the presence of their partner, how certain strategies worked, and statements about the perceived movement of the partner	“Es fühlte sich so an, als würde mich die Vibration suchen.” ”…um herauszufinden, ob sie wirklich sie ist.“ “Ich hatte den Eindruck, dass wir beide ähnliche Vorangehensweisen hatten … “
2.4.2.1 Finding / Identiying	Participants are certain that they have found or identified their partner, even if this is only for a short time. In some cases, specific phenomena are mentioned as justification	“The other one moved slightly and signaled to me where she was with small movements.” “I did think for a moment that I had met her …”
2.4.2.2 Being found	The feeling of being found by the other or finding each other	“…and then realized that the other person had apparently found me.” “I had the feeling that we both discovered each other immediately.”
2.4.2.3 Contact Quality	Different forms of sensing activity or presence, establishing connection, shared intimacy, synchrony, and mutual awareness	“…and finding our own communication.” “I experienced that we were trying to find each other.”
2.4.2.4 Contact Intensity	Contact intensity involves fluctuating presence, ranging from brief and faint to deep, strong, and enduring connections	“… very intensive contact.” “I didn't feel such a strong presence.”
2.4.2.5 Cooperation	Noticing or developing mutual tactics, coordinating movements, aiding each other, and feeling a collaborative connection	“I have noticed that we have gradually found a method to recognize “us” …” “Everyone adapts their pace to the other, taking care not to lose them …”
2.4.2.6 Transparency	The technical devices become transparent for social cognition and interaction, which is also expressed by metaphorical terms	“I was gradually able to look away from the alienating nature of the computer and meet my counterpart.” “…somehow like dancing together in a virtual space.”
2.4.2.7 Ambivalence	Participants are not certain that they have found their partner. Possible confusions with the static object or the shadow are conceded	“After that I was rather unsure.” “… but what if that was always her shadow?”
2.4.2.8 Cannot find	The partner cannot be found, or participants express that it is very difficult to find them	“When I couldn't find her …” “…which was quite difficult and unclear this time.”
2.4.2.9 Losing the other	Contact termination: the other avatar disappears or cannot be found again	“…that the partner had moved away.” “…until we have lost each other,”
2.4.3 Shadow	The shadow is explicitly or indirectly (but clearly) mentioned as the reference point of evaluation, including qualitative experiences	“I felt the shadow more often than the other player.” “The shadow was always at the same distance from her, but when I touched the shadow, she didn't seem to react to it …”
2.4.4. General Evaluation	Mixed experiences including control and self-assessment, strategy development, general success and failure, specific challenges, and fluctuating confidence. This code can refer to individual trials/rounds or to the entire experiment	“This round was strange and different to the others before it.” “Overall, I wonder whether the experiment is better solved with logic or with a good feel. I think it's a mixture of both.”
2.4.5 Learning Trajectory	Learning trajectories evolve from initial uncertainty to developing strategies and gaining confidence, but also include periods of doubt, reduced concentration, and fluctuating motivation	“You seem to get to know yourself and the space and everything better.” “I had the feeling that it worked pretty well at the beginning. In the middle of the experiment, it somehow became more difficult.”
3. MONADIC EXPERIENCE	*This category includes aspects which, although experienced in the dyadic setting, do not explicitly refer to it but rather to self-focused cognitive functions*	
3.1 Emotions	*All emotions are captured that occur during the experimental procedure. As a compromise between completeness and clarity, composites were formed from similar emotions*	
3.1.1. Positive		
3.1.1.1 Joy and fun		“I enjoyed it more and more.” / “It's funny.”
3.1.1.2 Relief and relaxation		“… which was a relief. “ / “I became more relaxed as the experiment progressed.”
3.1.1.3 Curiosity and excitement		“I felt exploratory, initially tentative, curious …” / “I found the experiment exciting at the beginning.”
3.1.1.4 Trust and Security		“…strong trust …” / “…which created a feeling of security.”
3.1.1.5 Connection and sociability		“I felt connected to the other person.”
3.1.1.6 Wonder and interest		“It was a surprising feeling …” / “It was very fascinating …”
3.1.2 Negative		
3.1.2.1 Uncertainty and confusion		“This created a feeling of insecurity. “ / “…but then I was like “disoriented”.”
3.1.2.2 Anger and frustration		“All in all, I'm annoyed …” / “There were signs of frustration.”
3.1.2.3 Disappointment		“I was also a little disappointed at times.”
3.1.2.4 Fear and worry		“…fear that we will lose each other again.” / “worry that I will be even more certain in the next moment.”
3.1.2.5 Isolation and loneliness		“I felt very isolated at the beginning “ / “…and lonely in the space.”
3.1.2.6 Dejection and disillusionment		“At the moment, I regret that there is no other way to communicate. “ / “I found this round sobering.”
3.1.2.7 Despair		“ … and had rather felt a sense of despair …”
3.1.2.8 Tension		“I became a little more tense …”
3.2 Rational consideration	Methodical or speculative thinking about the experiment, especially patterns and strategies, based on logical reasoning and observations	“…because I can't remember what it looked like during the experiment, whether the shadow was to the left or right of the person.” “… I wonder if she's noticed that too…”
3.3 Imagination	Visualizing the virtual space, the other person and their movements, creating a detailed mental image of the entire situation	“… visualize the room and the other person …” “I always had C's face in front of my eyes and how she moves on the imagined large disk.”
3.4 Concentration / attention regulation	Maintaining or losing concentration, focusing inward or on the other person, managing distractions	“…concentrated on the location of the static object in order to locate it.” “…strong inward turning, hardly any perception of anything else …”

Level 2	Description	Examples
DYADIC MICROACTIVIES	*Level 2 codes focus on dyadic micro-activities in a top-down manner, as they have already been described and used in former studies on social interaction or cognition*	
1. PROACTIVE-FOCUSING (A) – PF-A	Person (A) engages in proactive gestures that approach, touch, and follow their partner (B) to signal presence and initiate communication. This activity is experienced as originating from A and focused on B, partly in an offensive, provoking, or self-assertive manner taking the lead	“I approached them ‘nudging’ …” “I then moved back and forth, communicating with her.” “…to show that I am there and not the shadow or the object.”
2. RECEPTIVE-OPENING (A) – RO-A	Person (A) waits for, opens to, identifies, and accepts what is coming from their partner (B) as described with PF-A; an attentional space is given for what emanates from person B and is or could be perceived receptively	“I didn't move this time and waited …” “I tried to let myself be found instead.” “…tried to sense where the other person might be …”
3. PROACTIVE-FOCUSING (B) – PF-B	Analogous to PF-A with roles reversed; from the perspective of person A, proactively focused activities emanating from person B are perceived; one might feel addressed, touched, searched for, or followed. This activity can be experienced as a challenge or guidance by the other person	“I had the feeling that my counterpart was looking for me.” “… and when I moved away a little, she came right back.” “The other person moved slightly and signaled to me where she was with small movements.”
4. RECEPTIVE-OPENING (B) – RO-B	Analogous to RO-A with roles reversed; person A experiences their partner B opening to and receptively receiving what emanates from themselves (A); person A feels found, noticed, recognized. In addition, a weaker certainty about this activity, which emanates from the other person, also belongs to this category	“When I was found …” “It depended on my teammate's reaction to the vibration.” I had the feeling that the other person was waiting for me

To safeguard the qualitative analysis as well as subsequent quantification and statistical tests, intercoder reliability (ICR) tests were conducted covering almost all categories at different levels. For the four codes of “behavioral setting and embodiment” (Level 1), we refrained from using ICR tests, as their application in the coding of individual words is largely obvious (see above) or they occur very rarely and are therefore of little relevance (e.g., “body”). Depending on the number of codes to be tested together (four to fourteen), between hundred and hundred-thirty data segments were randomly selected from all coded segments. Both the selection of the segments from the individual codes and their arrangement in the test table were randomized and blinded before being independently reassigned to the categories by coders who were not involved in the project. After this first test, the level of agreement was calculated by Cohen’s Kappa and coding deviations were identified before conducting feedback sessions with the coders to further improve coding consistency (Campbell et al. 2013; O’Connor and Joffe 2020). In the feedback sessions, all data segments coded differently by the first coder (JW) and the other coders were discussed in view of the code definitions and test instructions leading to one of the following options: (a) argumentative agreement on one of the two coding variants, (b) double coding of the segment (according to both categories), (c) splitting the segment and partial coding, (d) no agreement. The results of the feedback sessions were incorporated into the coded data regarding both the individual segments and the implications for the individual codes drawn from the negotiation.

The ICR test procedure was conducted with six external coders for eight code sets at different hierarchical levels. All these coders were not involved in the study and its goals and not familiar with the PCP but received information about the experimental setting, devices, and procedure including participant instructions and partial excerpts from the codebook. Tests resulted in Kappa values ranging from of κ_1_ = 0.67 (substantial or moderate) to κ_1_ = 0.96 (almost perfect, *M* = 0.81) before and κ_2_ = 0.98 to κ_2_ = 1.00 (perfect agreement, *M* = 0.99) after the feedback sessions (Landis and Koch 1977; McHugh 2012; see Table 2). In sum, the ICR test results indicate the robustness and applicability of the category system. Based on that, in the next steps, the coded data were qualitatively interpreted and quantified to be further processed in descriptive and inferential statistics. In the case of multiple statistical tests referring to a specific configuration of variables, we assessed significance related to an adjusted critical alpha level according to the false discovery rate (FDR) control (Benjamini and Hochberg 1995), which appears appropriate for our explorative study (Glickman et al. 2014).

**Table 2:** Intercoder Reliability Tests

ICR Test (Number of Codes)	Discriminated Codes	K1	K2
Action Phases (4)	Motivation / Intention / Action / Evaluation	0.67	0.99
Intention (9)	Static Object / Partner Avatar / Explore behavior & space / Get in touch (active) / Get in touch (passive) / Keep in touch & follow / Help the other / Intuition / Shadow	0.85	0.98
Action (13)	Static object / Partner avatar / Searching / Change of direction / Forms of movement / Following & returning / Signaling / Interaction / Joint movement / Not or timidly moving / Moving away / Shadow / Insecure behavior	0.75	0.99
Evaluation (5)	Static object / Partner avatar / Shadow / General evaluation / Learning trajectory	0.72	1.0
Evaluation (10)	Partner avatar / Finding / Being found / Contact quality / Contact intensity / Shared strategy & cooperation / Transparency / Ambivalence / Cannot find / Being left & losing the other	0.81	1.0
Monadic Experience (4)	Emotions / Rational consideration / Imagination / Attention regulation	0.96	1.0
Emotions (14)	Joy & fun / Relief & relaxation / Curiosity / Trust & security / Connect. & sociability / Wonder & interest / Uncertainty & confusion / Anger & frustrations / Disappointment /Fear & worry /Isolation & loneliness / Sobering & bored / Despair / Tension	0.93	1.0
Microactivities (4)	Proactive-focused (A) / Receptive-opening (A) / Proactive-focused (B) / Receptive-opening (B)	0.80	0.98

Quantification of the qualitative codings was done in different variants. First, code frequencies based on the number of coded segments per category and participant were used. Second, these code frequencies were binarized so that only the information whether a category was applied to a participant’s data or not is retained. Third, for the subcategories or ‘code families’ of the action phases (Level 1) and microactivities (Level 2), the overlapping segments of these codes were identified for each participant to assess their interlevel or axial dependencies (Corbin and Strauss 1990). Fourth, all these measures were not only analyzed for the individual participants but also for the dyads in which case we can speak of “shared codes” (binarized) or the amount of a participant’s data that fit into categories under which the partner’s data are also coded (segment level). While binarized code frequencies are suitable for chi square tests, code frequencies at the segment level can be processed with ANOVA and t-tests.

An additional variant of quantification was applied to emotion words, since they can be operationalized in terms of valence and arousal (Russell 1980). To obtain concrete values for the emotion categories of this study, we compiled the results of three affective word rating studies in German language (Hepach et al. 2011; Schmidtke et al. 2014; Võ et al. 2009) providing comprehensive data bases. In this way, we considered the specific cultural context of emotion words (Mesquita and Frijda 1992; Gendron et al. 2014) and addressed the issue of generalization by averaging. In view of the task, context dependency is minimized, as all reference studies have no behavioral task context beyond assessing single words. From the databases of these studies, valence and arousal values of the emotion words corresponding to our emotion categories were taken and averaged. The code frequencies of our emotion categories were assigned to these points in the valence-arousal space, enabling a quantitative analysis of the emotions reported in the self-reports in the valence-arousal space both in a cumulated way and at participant level.

According to our observational approach, microactivities (Level 2, dependent variables) were analyzed regarding their coding overlap with and distribution across action phases (Level 1, quasi-independent variables). This is not only an extension of intralevel to interlevel code relations but also lends itself to investigating if there are any statistical differences between specific aspects of the microactivities (e.g., productive-focusing vs. receptive-opening). An initial finding leading to this idea was that the data partitions coded with microactivities (all forms cumulated) are dominated at Level 1 by the three most relevant action phases (intention, action, evaluation), while other codes only play a marginal role (Fig. 10). We therefore focused on these three action phases and determined the absolute code frequencies of PF-A, RO-A, PF-B and RO-B that fell under them for each data set (participant). To compare to what extent productive-focusing and receptive-opening microactivities depend on the action phases, code frequencies of PF-A and PF-B vs. RO-A and RO-B were added (PF = PF-A + PF-B; RO = RO-A + RO-B) and subjected to a two-way repeated measures ANOVA. With this factorial model (2 score types x 3 action phases), main effects and general interaction between PF and RO can be tested. To test specific differences between PF and RO across the action phases, two-tailed dependent-samples t-tests were conducted.

## Results

Starting with some aspects of early quantification of the text data, the protocol length (accumulated over the last six trials) ranged between 197 and 1,014 words (*M* = 543.5; *SD* = 195.8). The mean proportion of first-person singular pronouns (I, my, me) was 10.2% (*SD* = 2.6), and the corresponding value for plural pronouns (we, our) was 1.0% (*SD* = 1.0). In comparison with the proportions of first-person singular (5.0%) and plural (0.7%) pronouns averaged over different text genres (Pennebaker et al. 2015), the former value from our study is clearly higher. This indicates an increased attentional focus on self-referential introspective observation (Rude et al. 2004), from which it can be that participants were indeed following instructions to report on their own experiences and actions rather than describing other issues (e.g., assumptions about the purpose of the experiment etc.), thus speaking for methodological validity of the qualitative data. In contrast, the found proportion of first-person plural pronouns does not stand out strongly enough to allow for specific conclusions about we-consciousness or shared intentionality at a linguistic level (Goddard and Wierzbicka 2021; Vasil et al. 2023).

**Fig. 3:**
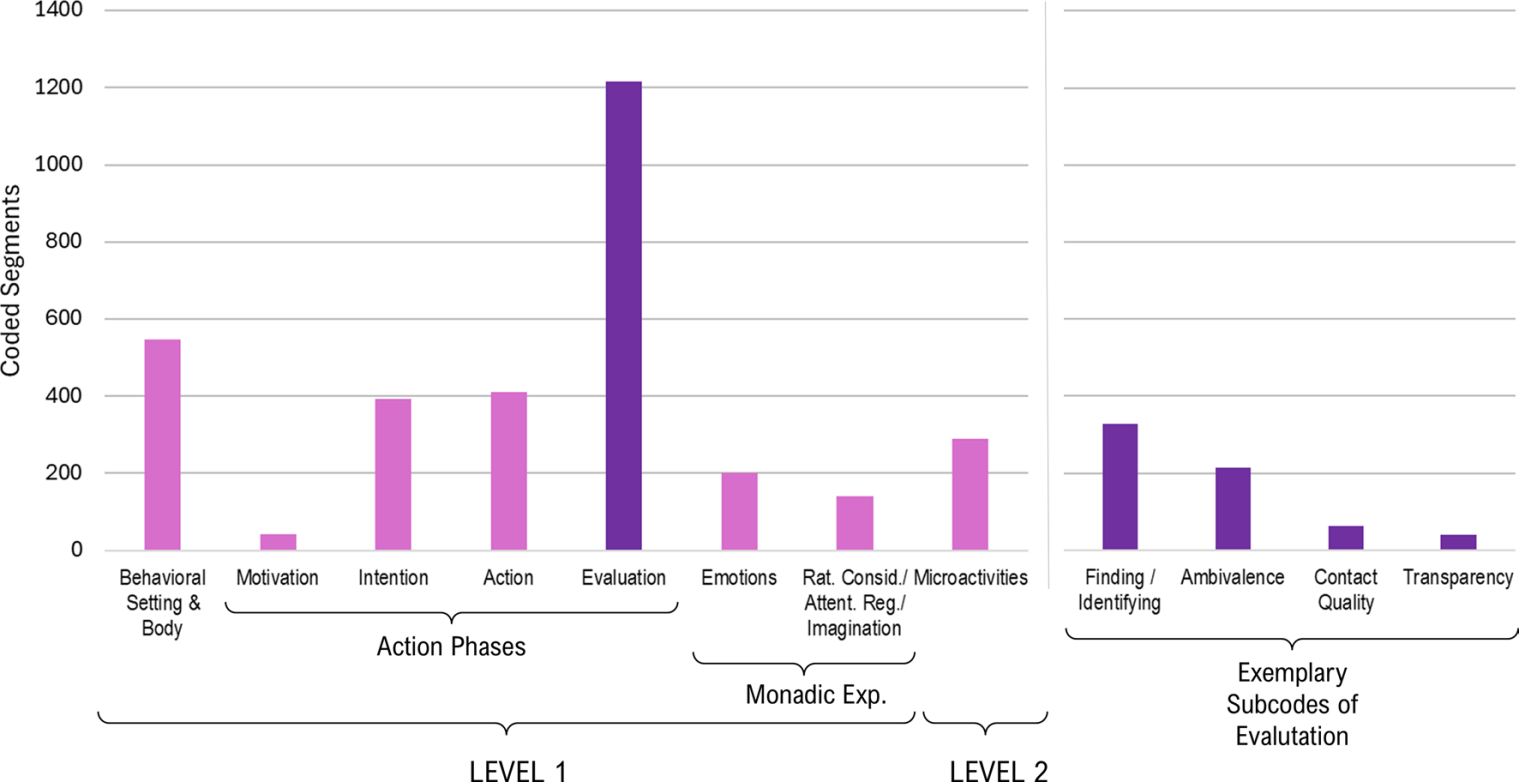
Distribution of coded segments

The qualitative analysis of the protocol data led to sixty-two (sub-) codes organized in two levels of a hierarchical category system (Fig. 2) with a corresponding codebook (Table 1). The distribution of the coded segments (Fig. 3) shows the action phases as the main topic with a clear focus on evaluation, while intention and action are in the midfield and motivation is only weakly pronounced. The latter can be explained by the fact that pre-decisional aspects of motivation (deliberating and choosing) were barely relevant in the context of the instructed task and participants’ compliance.

While agency represents the first-person complement to externally measured behavior, the next topic with decreasing frequency is behavioral setting & body reflecting the immediate nexus between these two perspectives. However, this category in itself does not play such an important role analytically, as the codings that dominate it quantitatively (vibration, button press, see Fig. 2) are mostly based on single words and therefore do not contain any additional information about subjective experience. For this, the context of neighboring categories in the data will have to be examined. Apart from this, the other two subcodes are only weak (test setting) or almost non-existent (body), whereby the latter is to be discussed below in the context of embodiment. Suffice it to say that only two segments coded under “body” refer to positive embodiment (focus on hands or fingers), while seven segments express an exclusion of sensory experiences (closed eyes). Codings pertaining to the test setting are mostly about time experience during the trials or the sequence of the various test phases.

**Fig. 4:**
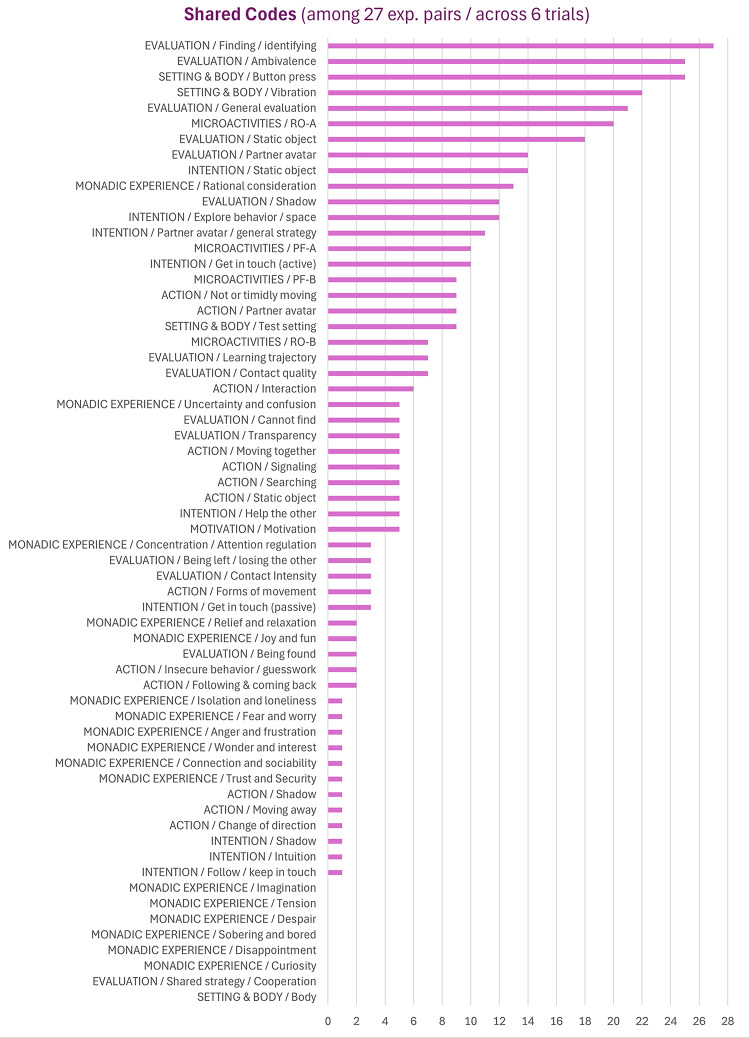
Shared codes.

The two remaining categories, monadic experience and microactivities, are roughly equally pronounced, but must be distinguished in that the former makes up the rest of Level 1, while the latter belongs to Level 2 as being superimposed to Level 1. Another difference already implied in the designation of “monadic” experience is their relevance in terms of dyadic sharedness. While the subcodes of monadic experience tend to be found in the lower ranks of the shared codes diagram (Fig. 4), the four microactivities are among the first third. Nevertheless, regarding monadic experience, we will examine emotions in detail, since they constitute an important cognitive dimension of subjective experience.

**Fig. 5:**
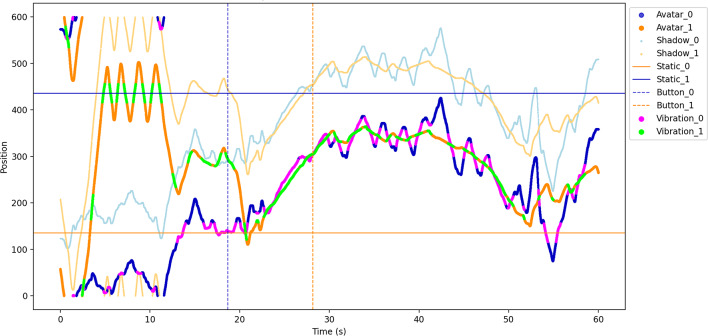
Movement trajectory over time with vibration and button press events (example). On the verticle axis, the position of the various objects is measured in an interval from 0 to 600, which represents the entire virtual circle.

Taking a closer look at the shared codes diagram (Fig. 4), it is not surprising that the most common codes associated with general task performance at an individual level also occupy the top positions of shared codes, simply because they occur in almost all data sets. More closely, focusing on evaluation as the top category, this is about the experience of *finding the partner avatar* or an *ambivalence* in this regard. In contrast, apart from the microactivities already mentioned, key phenomenological aspects of dyadic experience and interaction can be found in the middle of the diagram in *contact quality* and *transparency*. Exemplary results for these four codes, which are based on a purely qualitative analysis, are showcased in the following section. Subsequently, we focus on different results derived from late quantification of code frequencies, as indicated in this overview (Fig. 5).

### Qualitative aspects of evaluation (Level 1)

As mentioned, the codes of finding/identifying the partner avatar and ambivalence lead the ranking of both individual code frequencies (Fig. 3) and shared codes (Fig. 4). To begin with *finding/identifying* (327 segments from 54 data sets), this includes explicit statements of finding (“… I have found her …”) and respective certainty (“ … I was sure …”) but also a variety of more implicit expressions referring to the partner avatar: sensing/feeling, perceiving, noting, joining, encountering, meeting, coming around, being together, crossing paths, correctly identifying, making/having contact. In contrast to aspects of perception and sensing (“…at some point I thought I could feel her”), forms of logical reasoning must be distinguished (“As I couldn’t find anything when I searched further, I knew that this … must be my partner”). Another distinction can be made between self-centered formulations (“… I have found her …”) and those involving the partner avatar’s action (“moving towards me of the other”) or bidirectional interaction (“When I met the avatar, there was an immediate interaction”), all of which already reveal aspects of underlying microactivities.

*Ambivalence* (214 segments from 52 data sets) can be differentiated into four sub-aspects that can be related to those for finding/identifying. While finding/identifying occurs in the same trial for 38% of segments together with button press, the latter seems to be even more connected with ambivalence (43%), although this difference was not statistically significant, χ^2^(1, *N* = 54) = 1.47, *p* = .23., w = 0.1 Here are some examples of ambivalence with different aspects of button press: “The contact points were too short to press compared to the other times”, “On one or two occasions, I ‘regretted’ [pressing the button] because I thought I had found the person later”, “I always pressed the button, sometimes out of exuberance, and tended to press it too early without being sure … usually repeated vibrations in different places made me do it”. Analogous to the self-centered expressions mentioned above, ambivalence also includes self-reference with regard to participants’ metacognitive attitude (“… it was more like guessing”, “I didn’t want to judge too quickly”) or certainty about the result (“… questioning whether I really always find the person”, “Sometimes I wasn’t sure whether my perception of an interaction taking place wasn’t an illusion and the events were initiated by chance”). Complementarily, there are data segments pertaining to the partner avatar or the other objects: “I came across an element that had to be either my partner or her shadow”, “My counterpart was often close to an obstacle [the static object]”), “But then I thought to myself that it could also be my partner who just wasn’t moving”. Last not least, a lack of response or interaction was formulated: “… no clear, constant or stronger interaction”, “… not quite sure if she knew it was me or my shadow”, “after a series of attempts without confirmation, the feeling of insecurity has grown”.

*Contact quality* (62 segments from 27 data sets) was much less frequent than finding/identifying and ambivalence but goes beyond them in terms of additional experiential qualifications. Here, we can distinguish between more static and dynamic/interactional qualities, the former of which read, for example “I felt the presence of my partner”, “These moments felt warm and safe”, “I really had the feeling of a deep encounter and connection with my partner”, “a moment of timelessness, intimacy, closeness and harmony”, “I have experienced that there is a thinking person in the circle with me”, “I had the feeling that we were both sure it was the other one”, “In the end, I felt sympathy for the other person”. In contrast, dynamic qualities include formulations about alternating proximity and distance or connection and separation, as such as “I experienced that we were trying to find each other” or “it felt more like a joint exploration”. Finally, there are also few expressions of ambivalent or negative contact qualities: “brief shadowy encounters”, “It felt a bit empty”, “However, I had the impression that she was a little unsure”. Like the two previous subcategories, a distinction can also be made in these examples between self-referential utterances and the inclusion of the presumed feelings or experiences of the other person, as in the last example mentioned. We will encounter these aspects again below in the context of microactivities.

*Transparency* (39 segments from 18 data sets) is again less frequent than contact quality but is of particular theoretical interest, as it extends the latter by explicit or implicit aspects of experience transcending the technical medium. First, sensory associations can be identified such as eye contact (“… it felt a bit like intense eye contact”) and touching each other (“… almost as if you were always touching each other”) or more comprehensive ones such as sharing a room (“… as if you were in a room together”) or having a conversation (“…I had the feeling that we were talking”). Second, other metaphors go beyond elementary sensory associations, the most frequent of which expressed the feeling of (blind) dancing together (6 segments in 4 participants, see Auvray and Rohde 2012; Declerck et al. 2009). Here are other examples: “Just as two different waves meet and continue to move on the same wavelength in harmony”, “we really went for a walk together for some seconds”, “we hugged for a long time”, “it’s like telepathy”, “on the long journey together”, “wave the flag”, “repeated ‘greeting’ of the avatar”, “I ‘run over‘ [the other]”, “like playing catch or hide and seek”, “First, you walk past each other, see each other and then you go back together and talk more. From a brief hello to a longer conversation”, “… sometimes also a ‘teasing’ of the other”. Third, transparency also contained explicit statements about the experience of transcending the technical medium: “The circular virtual space became a real space”, “You forget the technical devices you are working with; you are only in your feelings and in your head”, “The room becomes more vivid, more real. You hardly notice that you’re holding something in your hand, it’s as if you’re moving in the room yourself and I have the feeling that I can see the circle in front of me”.

### Agency and objects (Level 1)

A first major quantitative result reveals the distribution of participants’ agency across the three interaction objects. For this purpose, the segment frequencies of the three relevant action phases (intention, action, evaluation) were added per data set (participant) for each of the three objects (static object, shadow, partner avatar) to introduce an object-dependent agency measure. This agency measure (in segments per data set) resulted in increasing values from the shadow (*M* = 2.4; *SD* = 2.9) over the static object (*M* = 5.2; *SD* = 4.2) to the partner avatar (*M* = 29.2; *SD* = 11.8). To interpret the agency measures against the background of behavioral measures (e.g., button press frequencies/rates) from other PCP studies, we compared them between the two moving objects (added segments of partner avatar and shadow, *M* = 31.6; *SD* = 13.4) and the static object with a two-tailed one-sample t-test and found a significant difference with large effect size, *t*(53) = 15.2, *p* < .001, *d* = 2.0. Also, the internal difference between the two moving objects was significant with large effect size, *t*(53) = 17.8, *p* < .001, *d* = 2.3, allowing for a robust discrimination of agency between the partner avatar and the shadow.

### Emotions (Level 1)

**Fig. 6:**
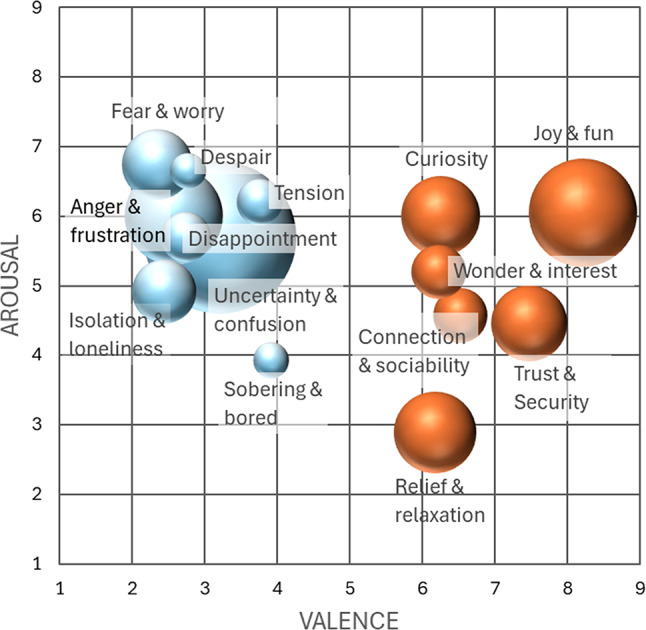
Emotions in the valence-arousal space. Score in the valence-arousal space were derived from comparisons of the coded emotions with three German affective word rating studies (Hepach et al. 2011; Schmidtke et al. 2014; Vo et al. 2009). The sizes of the bubbles relate to the code frequencies.

In contrast to the strongly pronounced action phases, emotions cover only 4% of the data and cannot be directly correlated with the behavioral measures in our perceptual crossing setting. However, the fourteen emotion codes provide fine-grained, hierarchically structured (positive/negative) aspects of participants’ experience during the task performance, which are presented here in the valence-arousal space based on the analytic procedure described above (Table 3; Fig. 6). While positive and negative emotions appear to be roughly balanced at first glance, negative emotions are slightly more pronounced (*M* = 2.1, *SD* = 2.9) than positive emotions (*M* = 1.6, *SD* = 1.9), although the difference is not statistically significant, *t*(53) = 1.5, *p* = .073, *d* = 0.2. Apart from this, negative emotions are slightly more differentiated (eight codes) than positive emotions (six codes), but the former tend to cluster around the most frequent code (uncertainty and confusion), and the latter seem to be more loosely distributed, though this remains quantitatively inconclusive. Furthermore, the two groups of positive and negative emotions can be interpreted as offshoots of the debated V-shaped or parabolic distribution of affective states in the valence-arousal space (Kuppens et al. 2013; Yik et al. 2023).

**Table 3:** Valence and arousal values for coded emotions.

Code	Frequency	Valence	Arousal
Joy & fun	28	8.21	6.05
Relief & relaxation	15	6.18	2.89
Curiosity & excitement	15	6.25	6.00
Trust & Security	14	7.47	4.46
Connection & sociability	6	6.52	4.57
Wonder & interest	8	6.23	5.20
Uncertainty & confusion	56	3.22	5.69
Anger & frustration	23	2.58	6.00
Disappointment	6	2.75	5.70
Fear & worry	13	2.35	6.74
Isolation & loneliness	10	2.44	4.91
Dejection & disillusionment	3	3.91	3.93
Despair	3	2.77	6.65
Tension	4	3.76	6.20

**Fig. 7:**
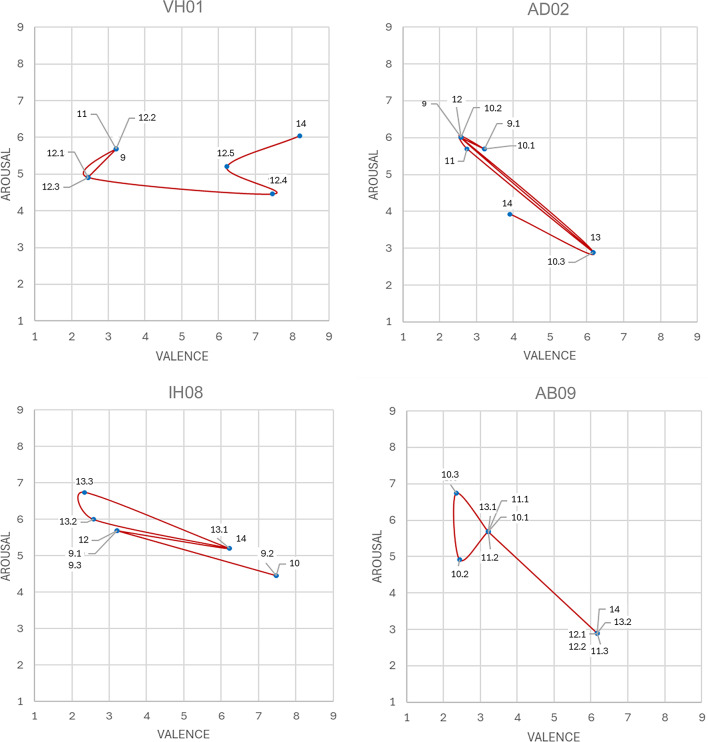
Individual emotion trajectories (examples). Lines were constructed by connecting the emotions found in the different trails and in sequences within individual trails according to their numbering, so they represent the chronological order of the emotions felt.

Since this multimethod analysis of emotions is based on participants’ accumulated self-reports, it can also be used to examine the individual diachronic structures of emotions over the course of the trials. To this end, for each participant the emotion codings were located in the valence-arousal space and ordered in their temporal sequence (as occurring in the protocols) leading to individual trajectories examples of which are shown in Fig. 7. However, it should be noted that the participants expressed a rather fluctuating number of emotions or even none at all, so it was not possible to obtain such trajectories for all participants.

### Dyadic microactivities (Level 2)

**Fig. 8:**
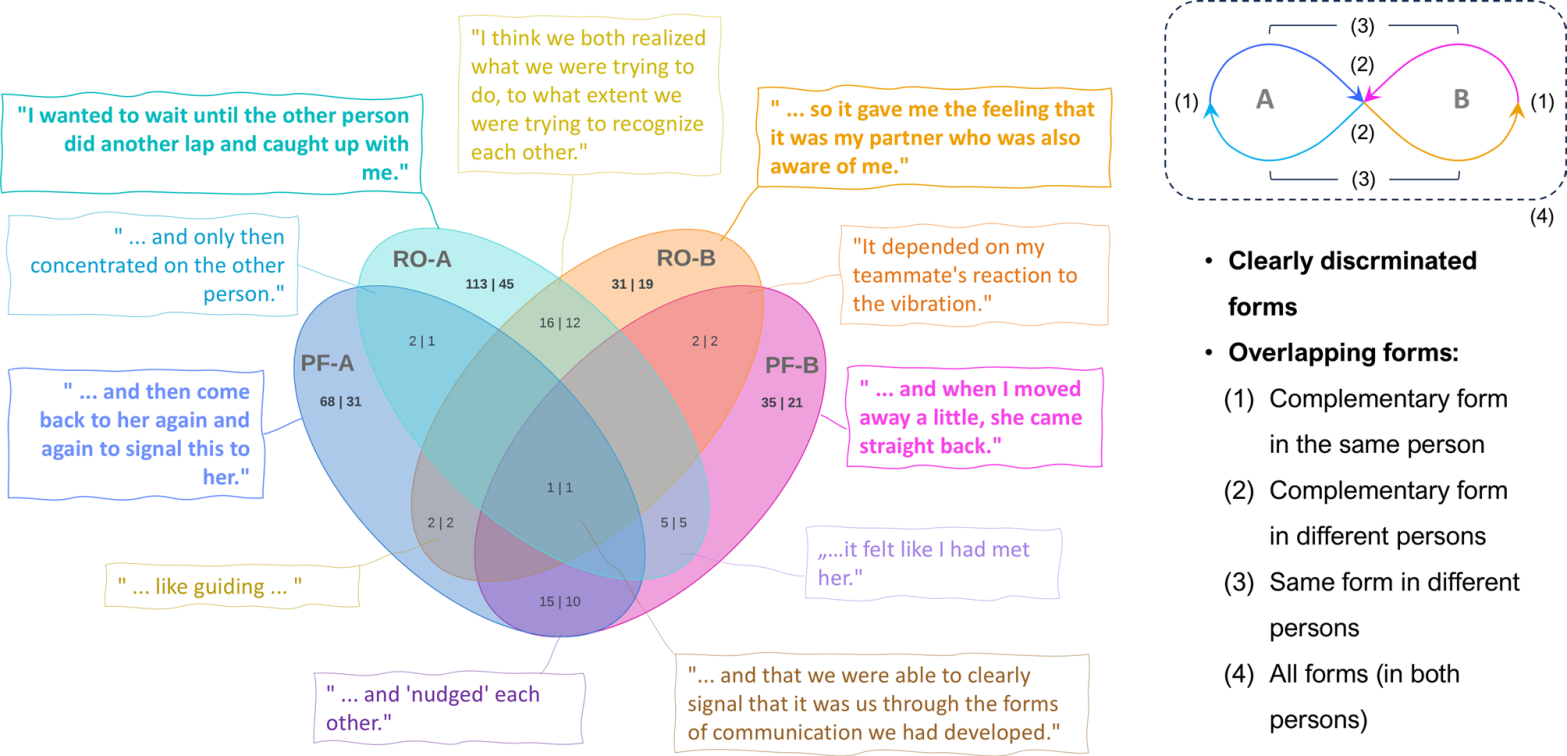
Dyadic microactivities: Intralevel code-relations with data examples. In the Venn diagram (left) the overlapping areas represent the coding overlaps of two or more categories, while non-overlapping areas represent the codings of clearly distinguished categories. The first number in the fields shows the number of coded segments, the second one shows the number of data sets including this category or combination of categories. Empty fields mean that mean that there is no data coded according to this specific combination. The lemniscatic diagram (above right) represents the complementary relationship of the four microactivities, arrow directed toward the interaction partners refer to proactive-focusing activity, and arrows pointing behind the interactants refer to receptive-opening activity

As indicated above, two code sets were distinguished, the first of which includes overlaps between the four microactivities) resulting in 290 segments while the second only comprises clearly discriminated microactivities (247 segments, see Fig. 2). To illustrate the distribution and overlaps of the microactivities in these code sets, Fig. 8 explains this with data examples. Below, we start with the clearly discriminated microactivities to compare their binarized code frequencies including results from former studies, then we use the larger, less differentiated code set to obtain a general overview of interlevel code relations for the current study and finally return to the clearly discriminated microactivities to examine specific code combinations with statistical tests.

**Fig. 9:**
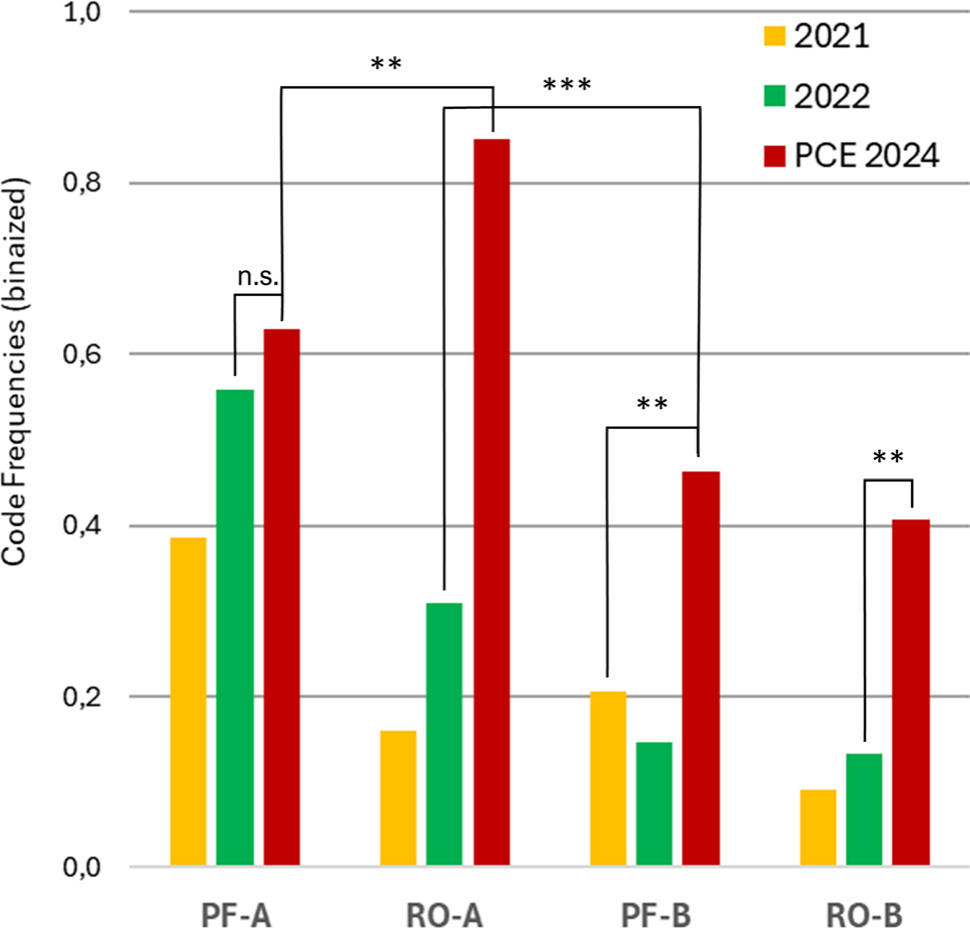
Microactivities across experimental settings. Differences of binarized and relative code frequencies computed with chi-square tests, **p < .01, ***p < .001

In addition to the significance of microactivities for this study, as mentioned above regarding their ranking with shared codes, they build a bridge to other first-person studies on social cognition conducted by the first author. Comparability between the studies can be justified by the following points: (1) Despite different settings (group, dyadic) and modified tasks, the qualitative questions were the same and the recording conditions were very similar, as in all studies participants were asked to write down what they experienced and what they did. To achieve comparability, the questions in our study were formulated in the same way as in the other two. (2) The code definitions of the microactivities are almost identical, slightly different wordings are due to the specific task/setting which, however, does not affect the character and structure of the microactivities. (3) For the cross-study comparison only binarized code frequencies were used minimizing task-specific differences of the protocols. – Compared to more direct or natural settings of social interaction in the group experiment with changing dyads (Wagemann & Weger 2021) or stable dyads without and with face masks (Wagemann et al. 2022), we assumed that microactivities would not be very pronounced in the technologically dominated setting of the PCP paradigm. However, quite to the contrary, all four microactivities were more frequent among participants than in the other two studies and three of them (RO-A, PF-B, RO-B) were even statistically significantly higher than the next smaller values of the previous studies (Table 4; Fig. 9), even taking into account an adjusted critical α-level due to multiple testing (6 tests, Benjamini and Hochberg 1995). Within the current study, RO-A was significantly higher than the other three microactivities.

**Table 4:** Level 2: microactivities across experimental settings

	RO-A: 2024 vs. 2022	RO-B: 2024 vs. 2022	PF-B: 2024 vs. 2021	2024: RO-A vs. PF-B	2024: RO-A vs. PF-A
*M*_*1*_	0.852	0.407	0.463	0.852	0.852
*M*_*2*_	0.309	0.132	0.205	0.463	0.630
*p*	< .001	.006	.008	< .001	.008
*χ* ^*2*^	*χ*^*2*^(1, *N*_*1*_ + *N*_*2*_ = 88)		*χ*^*2*^(1, *N*_*1*_ + *N*_*2*_ = 98)	*χ*^*2*^(1, *N* = 54)	
	26,8	7,5	7.2	18,1	6.9
*w*	0,55	0.29	0.27	0.41	0.25

M_1_ refers to the first and M_2_ refers to the second of the compared conditions. Only significant results are shown.

**Fig. 10:**
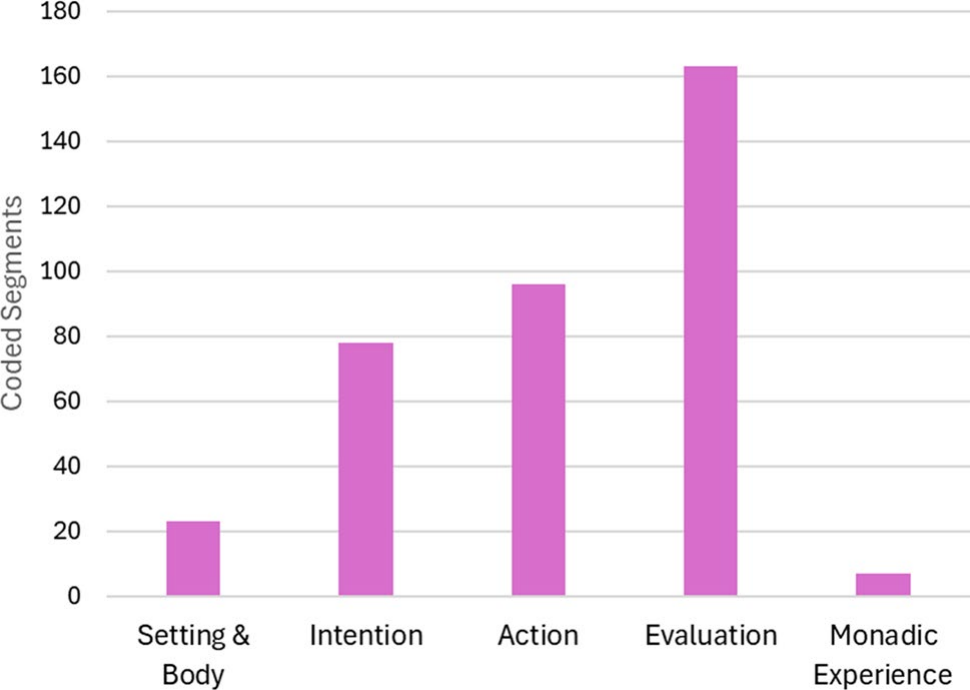
Dyadic microactivities: Interlevel code relations (general). Cumulated microactivities (all forms including intralevel overlaps) coincide with data coded according to level 1 categories, as depicted.

**Fig. 11:**
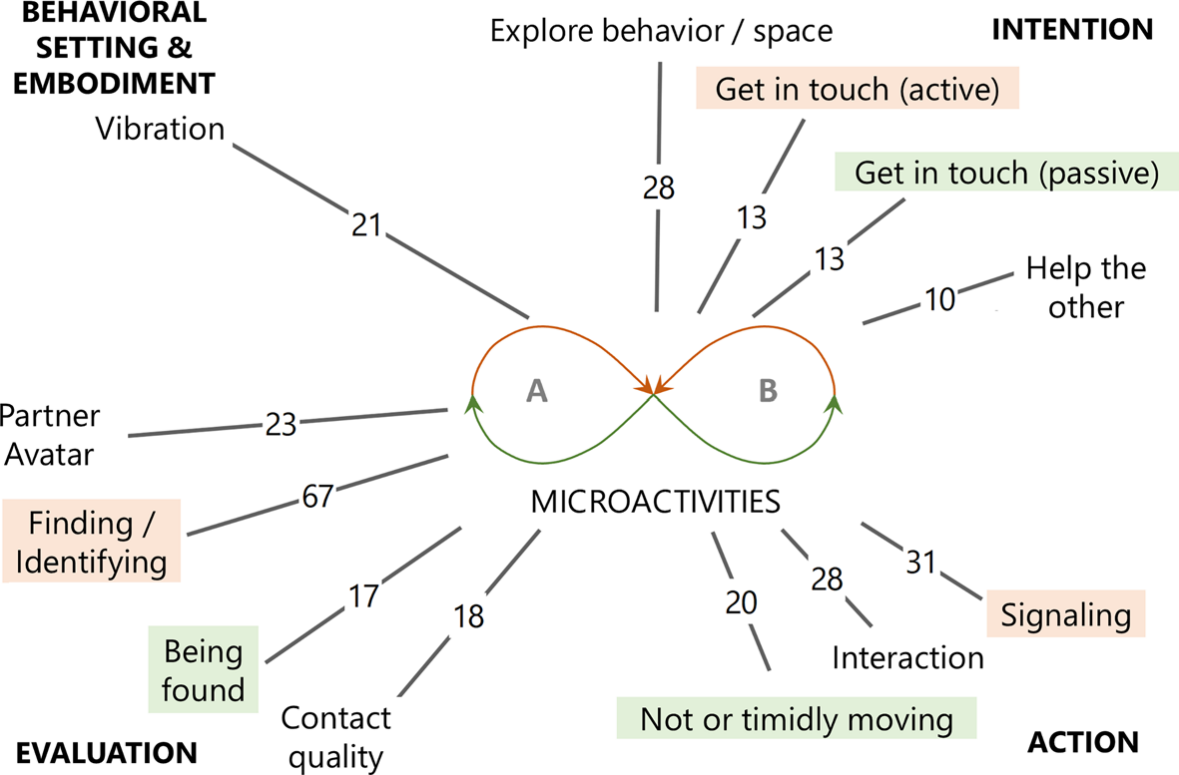
Dyadic microactivities: Interlevel code relations (detailed). Microactivities, indicated with a lemniscatic diagram in the center (see Figure 8), make up the reference point for surrounding Level 1 categories. Numbers indicate how many data segments are double coded with respective Level 1 categories and microactivities (minimum number of overlapping segment: 10). Corresponding action phases with a predominantly proactive-focusing (red) and receptive-opening (green) character are highlighted.

To delve deeper into the current study, we report on the Level-1 coding background of microactivities or, respectively, their interlevel overlaps based on the less differentiated code set. Figure 10 shows that microactivities are most strongly associated with the action phases which is not surprising, as they represent specific forms of (social inter-)action establishing the content of the diachronic action phases. However, one could ask why there is also a small but present proportion of *monadic* experience underlying *dyadic* microactivities. This is primarily based on five segments of rational consideration that incorporate specific microactivities into broader reasoning or justification, which means that this cognitive category is monadic in nature, while its object of reference need not be. In Fig. 11, the four most frequent categories are broken down to those subcategories that essentially make up their contribution to the microactivities. A correspondence between proactive-focusing and receptive-opening aspects can be identified here and tracked across the action phases, which suggests checking the distribution of the respective microactivities (see method section). To examine the (non-causal) effects of action phase (intention, action, evaluation) and score type (PF, RO) on participants’ scores a two-way repeated-measures ANOVA was conducted. There was a significant main effect of action phase, F(2, 102) = 18.43, *p* < .001, partial η² = 0.27, indicating that mean scores differed across the three phases. There was also a significant main effect of score type, F(1, 51) = 14.17, *p* < .001, partial η² = 0.22, suggesting that PF and RO scores differed overall. Most importantly, the interaction between action phase and score type was significant, F(2, 102) = 9.79, *p* < .001, partial η² = 0.16, indicating that the difference between PF and RO scores varied by action phases. To break this global result down to the action phases, individual t-tests were conducted showing for intention almost no difference between PF (M = 0.67, *SD* = 1.0) and RO (*M* = 0.69; *SD* = 1.0), *t*(53) = 1.38, *p* = .9. In contrast, for action there was a significant difference with medium effect size between PF (*M* = 0.89, *SD* = 1.41) and RO (*M* = 0.46; *SD* = 0.64), *t*(53) = 1.87, *p* = .034, *d* = 0.39, as there was also for evaluation with large effect size between PF (*M* = 0.72; *SD* = 1.12) and RO (*M* = 1.89; *SD* = 1.83), *t*(53) = 3.98, *p* < .001, *d* = 0.77. Considering an adjusted critical α-level due to multiple testing (3 tests, Benjamini-Hochberg, 1995), the result for action becomes non-significant (α_crit_ = 0.33), while the result for evaluation remains significant. So, in sum, these results support a phase-dependent relationship between PF and RO, driven primarily by differences in the evaluation phase.

## Discussion

Of the many topics that could be discussed regarding the presented results, we will limit ourselves to two core aspects of enactivism, in the context of which the PCP was developed, namely interactivity and embodiment. To begin with the former, we can ask about the empirical relations of individual or monadic first-person experience and dyadic experience integrating the second-person perspective (Wagemann et al. 2022). While interpretation of monadic experience could be easily restricted to representative cognitive processes located in the isolated individual or even in neural processes, as put forward by Theory of Mind (Schaafsma et al. 2014; Shanton and Goldman 2010), evidence of dyadic or second-person aspects would support an interactive account of social cognition including direct forms of perception (Gallagher 2008; Krueger and Overgaard 2012). First, against this background, the purely quantitative analysis of singular and plural first-person pronouns seems to favor a more individualist framework, apart from confirming methodological validity of the qualitative data based on a high amount of first-person singular pronouns compared to other genres of text data. However, the linguistic preference for I-words over we-words in our data offers only one perspective on the above question and is qualified by studies that show the dependence of the use of first-person pronouns in singular vs. plural on cultural or societal background (Na and Choi 2009; Uz 2014). Considering the qualitative analysis of the evaluation category, our observation that both monadic and dyadic aspects can be found in all the subcodes presented calls for more subtle explanations than mere word counts. In fact, dyadic aspects – and thus phenomena of participatory sense-making (De Jaegher and Di Paolo 2007) – become increasingly concrete and qualitatively differentiated in the transition from the more frequent to the rarer codes (finding/identifying, ambivalence, contact quality, transparency), which suggests that they would elude superficial investigation. Transparency in particular is a decisive criterion by which tools in general but also mind-enhancing tools and sensory substitution devices such as the Perceptual Crossing device can be assessed in terms of their intended usability (Auvray and Myin 2009; Clark 2003). For example, the experience of dancing goes beyond an anecdotal character mentioned by Auvray and Rohde (2012) and is fascinating because it implies the interactive aspect of not only staying in contact with another person, but also moving together in different directions, back and forth, which is clearly confirmed by the behavioral data (Fig. 5, from about 20 ms). Together with the (less pronounced) category of Shared Strategy, the experience of really interacting with the partner and pursuing a common goal with them is similar to the phenomenon of joint agency including the “shift from a sense of self-agency to a sense of we-agency” (Pacherie 2014, p. 25). While, in our context, self-agency can be associated with the subcategories referring to the static object and the shadow, we-agency seems to occur with Transparency and Shared Strategy, bearing in mind, however, that the common goal lies in nothing other than dyadic interaction itself. Zooming out from the subcodes of evaluation, an interactionist view can be further strengthened by the high proportion (70%) of text data pertaining to shared codes. In terms of agency on a general level, the different types of objects also speak in favor of interactivity. At first sight, our results of object-specific measures of agency simply seem to confirm Auvray and colleagues (2009) finding that the three objects can be well distinguished based on the specific stimulation experienced by participants. It must be noted, however, that this effect disappeared for the proportion of clicks and stimulation (i.e., the object-specific percentage of clicks divided by the corresponding percentage of stimulation) regarding the discrimination of the two mobile objects (partner avatar, shadow). Additionally, stimulation cannot be equaled with agency, since the former is purely receptive, while the latter integrates proactive and receptive mindsets which will be further elaborated in terms of microactivities. Therefore, the first-personal view inside the black box goes beyond a combination of behavioral stimulus (vibration) and response (click or button press) measures in that it puts forward agency as an integrated proactive-receptive dimension of cognition providing a clear discrimination of all three objects from each other. While we agree with Auvray and colleagues’ inference from their results that participants succeeded in the task due to “their ability to favor the situations of mutual perception” (Auvray et al. 2009, p. 8), our findings make this interactive dimension explicit through the differentiated subcodes of the action phases.

After arguing for an interactionist interpretation of the initial results, monadic or individualistic aspects of cognition should also be included, without which the picture would remain incomplete, as also Di Paolo and De Jaegher (2017) note. Admittedly, regarding the small portion of data coded under monadic experience (10%), explicit evidence of individualistic aspects is clearly less pronounced than the rest of Level-1 categories exhibiting direct reference to interaction. But as in many debates with opposing positions, the solution here may also lie in an empirically based balancing of the two sides, without negating the predominance of one of the two, namely interaction. Having said this, our analysis of emotions as an example of monadic experience offers important insights into the personal mental life of participants and thus complements interactive cognition equally as agency does. In particular, the monadic character of emotions (4% of the data) can be explained by the fact that in the minimalist setting of PCP, all aspects of intersubjective or intercorporeal affectivity, which are mediated by visual (mimic, gestural, postural, kinaesthetic), auditory (voice), or body touch expressions of emotion by the other person (Fuchs 2013, 2017) are excluded per se. What remains is haptic stimulation by vibration the specific patterns of which, by means of transparency, can be realized and felt as emanating from the other person’s actions. Thus, considering different levels of intercorporeality according to the range and directness of sensory modalities included, participants’ emotions can only be partly traced back to intercorporeal resonance, but refer primarily to their own interaction experience and performance evaluation, which is much more indirect than full and immediate perception of the embodied person.

From a global perspective, the valence-arousal map shows clearly separable regions of negative and positive emotions with eight codes and a non-significant lead in segments per participant for the former compared to the latter with six codes. Considering the scarce data, this could be interpreted as a slight indication of the ontogenetically rooted negativity bias (Dunn 1988; Vaish et al. 2008) that here, however, does not refer to thematic aspects of the task, as in emotion research, but rather takes the role of a motivating drive or evaluative response referring to the fact or possibility of failing in the task (Proust 2015; Wagemann, 2020). Consequently, the presumed negativity bias can be assessed as methodologically beneficial here, as the risk of failure can lead to an increased introspective awareness of participants’ mental life and agency. While the negative emotions could spur participants to systematically improve their performance, positive emotions include desirable emotions with higher (e.g., joy and fun, curiosity) and lower arousal (relief and relaxation). Therefore, complementary to negative emotions, the spectrum of positive emotions can be seen as what intrinsically motivates participants in terms of a reward to be achieved (Isen and Reeve 2005; Løvoll et al. 2017).

The outlined interpretation of emotions can be substantiated at a single participant level, where more dynamic and individual signatures come into view. Despite the generally monadic character of emotions, the emotion trajectories (Fig. 7) can be understood as indirectly referring to the interaction dynamics between the dyadic partners fueled by driving (negative) and anticipated (positive) affective states. In particular, the trajectories show both minor fluctuations that remain in the positive or negative range, as well as far-reaching changes in between, the latter of which could be interpreted as transitions between situations of success and failure in finding the partner. The slope of the trajectories between the positive and negative states can be used to identify different characteristics, namely those that remain at one arousal level despite the shift in valence (flat slope) and those that assume lower arousals in positive states (negative slope). Note, however, that this is an explorative approach to emotions experienced during a perceptual crossing task, that needs to be further examined in follow-up studies due to the paucity of emotion data and the mentioned indirectness.

With dyadic microactivities, we return to an unambiguously interactionistic key dimension of perceptual crossing, as all of them equally refer to one’s own and the other person’s actions. The unexpected increase in all microactivities compared to more direct or natural settings (Wagemann & Weger, 2021; Wagemann et al. 2022; Fig. 9; Table 4) can be explained by the PCP setting, which not only does not seem to be a hindrance, but even a benefit. Exactly what participants prevents from affective intercorporeality with their partners, thus leading to monadic forms of emotion experience, might give room for an awareness of those experiential and agentive aspects of social interaction which otherwise tend to remain pre-reflective. In other words, the minimalist PCP setting excludes the usual multisensory stimuli that might otherwise distract participants’ attention from the more subtle, mental and physical microactivities through which they establish, maintain and playfully vary contact with their partner. Here, in contrast to the other settings, the partners are not co-present in an obvious way but must be found again and again, which explains the also unexpected significant increase in receptive-opening activity emanating from the reporting person (RO-A).

At this point, we extend the discussion to embodiment. Above we have mentioned that under the category of body, which might initially appear to be the closest, there are hardly any relevant experiences to be found (Fig. 2), and if so, then rather negative ones (eyes closed). As a next candidate, the action phases can be assumed to include information on embodiment, as scholars have emphasized the embodied nature of action (Gallagher and Zahavi 2008; Merleau-Ponty 1962). However, from a strict phenomenological perspective, only the explicit execution of actions (e.g., specific movements) and related sensory sensations (e.g., vibrations) can be regarded as embodied in our setting, but not intentions that prepare or accompany actions or evaluations that look back on actions performed. Although intentions and evaluations are certainly associated with brain activity in the agents (Agosta et al. 2011; van Timmeren et al. 2023), this does not justify speaking of them as phenomenologically “embodied”. At best, weak forms of embodiment can be admitted for intention and evaluation, since they are processually linked with physical action execution.

To avoid settling for an unspecific assertion that perceptual crossing involves embodied (inter)action in some way, we refer to previous first-person studies in which these topics have already been examined in more detail. First, with a general focus on the action phases, our art-based research study on moulding a hollow clay sphere (Wagemann & Starosky 2024) demonstrated that intention was significantly dominated by mental action (e.g., strategic consideration), while in the second phase physical action (e.g., specific processing of the material) was significantly stronger. In the evaluation phase, mental action (e.g., decision in process flow, acceptance of problems) was prevalent though this was not significant. In sum, these findings support the above idea that action phases can be associated with different grades or degrees of embodiment, as physical action is more, and mental action is less embodied in the phenomenological sense. Together with the finding of continuously alternating action phases during task performance, this can be interpreted as dynamic embodiment. Second, returning to microactivities, in our study on the impact of face masks on social cognition, PF-A/B turned out to be significantly more embodied based on the higher frequency of body-related words in the coded data compared to RO-A/B (Wagemann et al. 2022). Although this was found in a natural, multisensory setting, it can be transferred to the PCP in which PF-A/B are clearly tied to physical actions executed with the PC device, while RO-A/B are more independent of physical actions but rather realized via mental actions such as assuming the attitude of waiting for the other to be found.

**Fig. 12:**
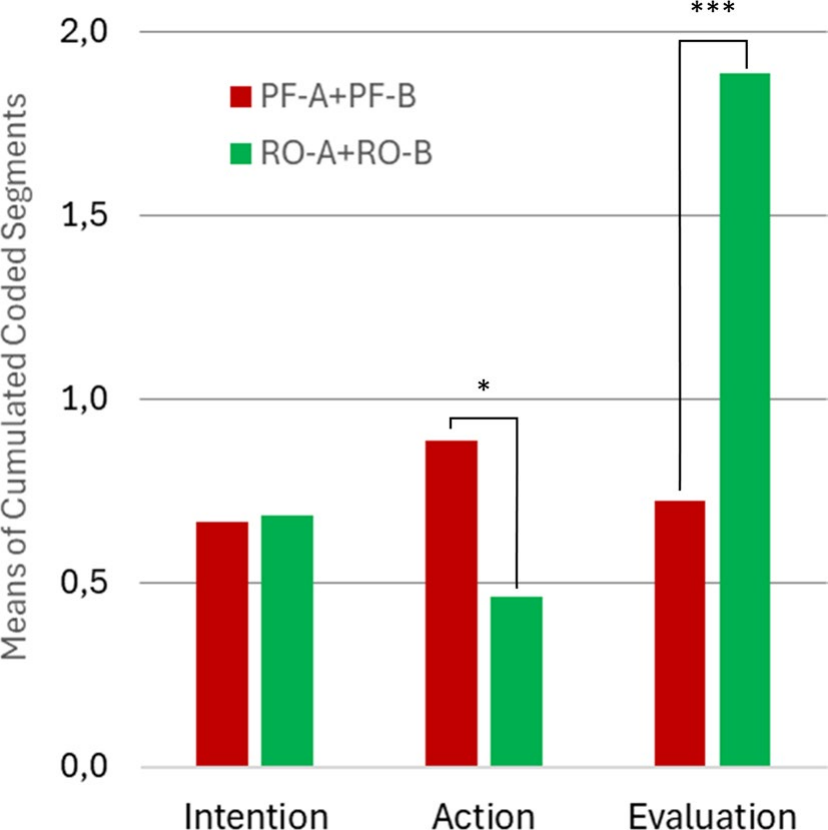
Dyadic microactivities: Interlevel code relations (detailed). Cumulated microactivities coincide with the action phases. * *p* = .038, *** *p* < .001. The difference for action becomes non-significant with a corrected critical alpha level (α_crit_ =. 33)

**Fig. 13:**
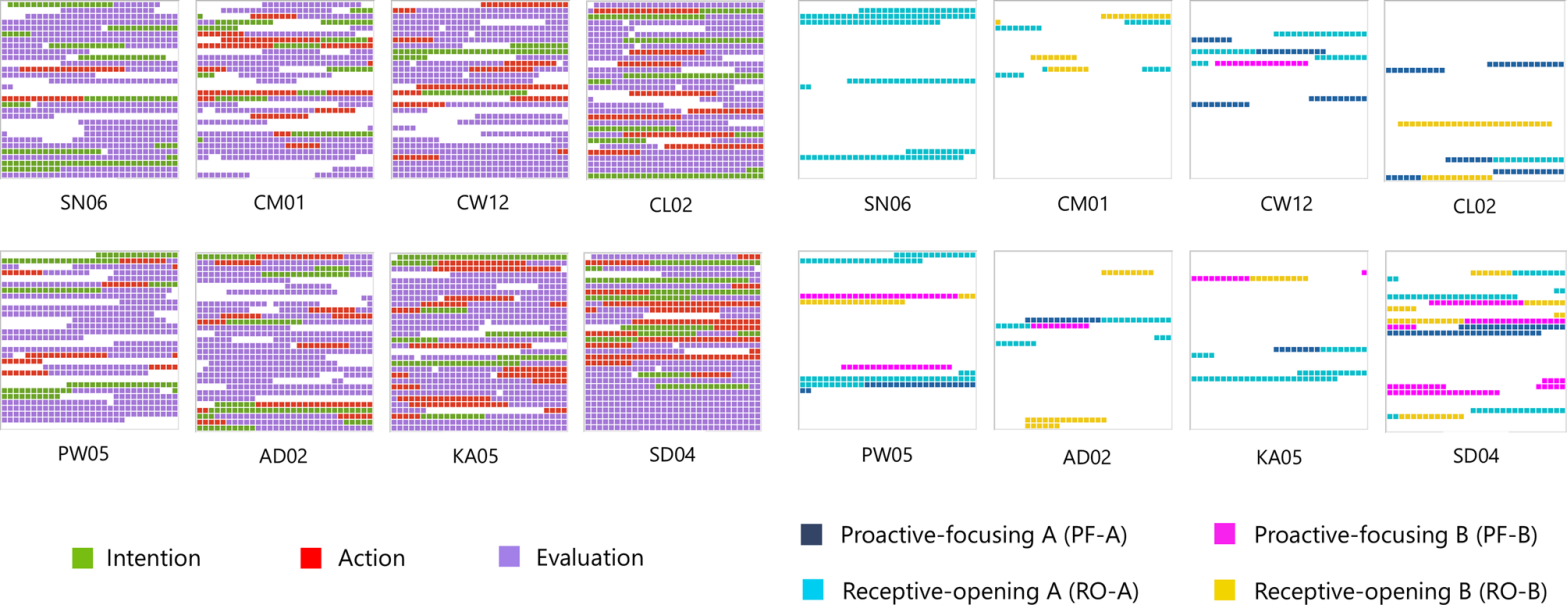
Embodiment dynamics of action phases and microactivities. 40 × 40-pixel squares represent normalized data sets with according proportions of codings. While the action phases (left) cover large parts of the data) (*M* = 78%), microactivities (right) are more sparsely distributed (M = 10%). Action phases and microactivities show individual patterns predominantly alternating between weaker (Intention/Evaluation, RO-A/B) and stronger (Action, PF-A/B) forms of embodiment

Against this background, different degrees and dynamics of embodiment can also be inferred here regarding the relations between the action phases and microactivities. Already from the interlevel analysis (Fig. 10), the strong connection between microactivities and action phases becomes clear and is further differentiated by specific subcodes bearing a proactive or receptive character (Fig. 11). In fact, the distribution of proactive-focusing (PF) and receptive-opening microactivities (RO) across the action phases shows a statistically significant pattern of interaction between PF and RO at a global level (see Sect. 3.4 and Fig. 12). Although this is primarily due to RO > PF in the evaluation phase, together with PF > RO in action execution a complementary dependence of PF and RO on the action phases can be assumed. In other words, even in this minimalist, sensory-deprived setting, graded forms of embodiment seem to be relevant. On the one hand, stronger experience of embodiment can be associated with physical action execution (second phase) and weaker embodiment is lived through in intention and evaluation (first and third phase), as suggested by the cited studies. Consequently, since both the action phases and microactivities periodically alternate during task performance (Fig. 13), we can speak of dynamic embodiment undergoing phases of less and more distanced experience of one’s body. On the other hand, the general level of embodiment in the perceptual crossing task is relatively low compared to more natural settings enabling multi-sensory perception and intercorporeal resonance.

## Conclusion

For drawing substantial conclusions about this study, it might be too early, as not all data types collected were considered here (except Fig. 5). Nonetheless, regarding the qualitative data and the multimethod analyses presented, we can speak of the unfolding of a rich and diverse landscape of first-person experience and subjective agency in perceptual crossing, which was previously unknown to this extent and at this level of accuracy. Already without including the other data, our findings partly confirm previous research on perceptual crossing and social cognition in the context of first-person methodology, but in part they also encourage a more differentiated and flexible view of enactivist key concepts such as interaction and embodiment. First, Auvray and colleagues’ (2009) finding of participants’ ability to discriminate the three interaction objects could be confirmed and even refined in terms of the two mobile objects based on the quantified amount of respective agentive experience. Second, in continuation of, but also in contrast to first-person studies on social cognition with more natural settings (Wagemann & Weger 2021; Wagemann et al. 2022), a high relevance of dyadic microactivities for perceptual crossing could be demonstrated. Due to the qualitative and quantitative susceptibility of microactivities in relation to empirical settings, their complementary dynamics can be understood as a mostly pre-reflective basic structure of social cognition, which, however, can be made conscious by introspection. Contextualizing ecological psychology with Steiner’s approach to social cognition that also inspired our first-person reference studies (Wagemann et al. 2022; Wagemann & Weger 2021), the interpersonal coupling of microactivities exhibiting both actional and perceptual features is an action-perception cycle (Gibson 1979), establishing an independent sensory modality for recognizing other human agents, i.e., the sense of I and Thou (Steiner 1966). Third, as supported by Di Paolo and De Jaegher (2017), the full scope of subjective experience in PC includes both dyadic/interactionistic and monadic/individualistic aspects. Moreover, our analyses and interpretations suggest going beyond a static concept of embodiment in favor of a graded and dynamic account integrating changes between stronger and weaker forms of embodiment. Not least the already debated integration of interactionist and individualist aspects in social cognition invites us to think of embodiment in an empirically open and multi-layered way instead of treating it as an unquestionable naturalistic dogma (Kirchhoff and Hutto 2016). Even among neuroscientists, graded approaches considering stronger and weaker embodied forms of cognition are discussed in view of the varying correlation of these processes with unimodal, multimodal, and amodal parts of the brain (Chatterjee 2010).

In methodological regard, our study strengthens a systematic and comprehensive use of qualitative first-person data, ties up with proven analyses comprising early and late forms of quantification, and introduces novel procedures, such as those for emotions and the statistical analysis of microactivities across action phases. Nevertheless, due to the restriction on qualitative data in this article and the exploratory character of the study, there are also some limitations. First, we are looking forward to the behavioral and personality data analyses and their triangulation with the results presented here, which may lead to further confirmation or questioning of the proposed interpretations. Second, testable hypotheses can only be formulated after further clarification and sharpening of the overarching results of this perceptual crossing study, which can then be investigated in strictly experimental follow-up studies. Last not least, further research could, for example, modify the setting and the instructions in such a way that a higher yield of relevant data regarding emotional experience could be expected, which would better substantiate the methods and possible interpretations outlined here. Until then, we regard the investigation and discussion presented here as a first step in this direction and as a stimulus for further research on social cognition substantiated by a mixed-methods and mixed-analysis first-person methodology.

## Data Availability

The raw data (in German language) generated and analyzed in this study as well as statistical data are available under https://osf.io/36us9/?view_only=f3793772172b4646b9fd5969e796377f.
